# Methodological challenges and potential solutions for economic evaluations of palliative and end-of-life care: A systematic review

**DOI:** 10.1177/02692163231214124

**Published:** 2023-12-23

**Authors:** Claudia Fischer, Damian Bednarz, Judit Simon

**Affiliations:** 1 Department of Health Economics, Center for Public Health, Medical University of Vienna, Vienna, Austria; 2 Ludwig Boltzmann Institute Applied Diagnostics, Vienna, Austria

**Keywords:** Economic evaluation, end of life care, methodology, palliative care, systematic review

## Abstract

**Background:**

Given the increasing demand for palliative and end-of-life care, along with the introduction of costly new treatments, there is a pressing need for robust evidence on value. However, comprehensive guidance is missing on methods for conducting economic evaluations in this field.

**Aim:**

To identify and summarise existing information on methodological challenges and potential solutions/recommendations for economic evaluations of palliative and end-of-life care.

**Design:**

We conducted a systematic review of publications on methodological considerations for economic evaluations of adult palliative and end-of-life care as per our PROSPERO protocol CRD42020148160. Following initial searches, we conducted a two-stage screening process and quality appraisal. Information was thematically synthesised, coded, categorised into common themes and aligned with the items specified in the Consolidated Health Economic Evaluation Reporting Standards statement.

**Data sources:**

The databases Medline, Embase, HTADatabase, NHSEED and grey literature were searched between 1 January 1999 and 5 June 2023.

**Results:**

Out of the initial 6502 studies, 81 were deemed eligible. Identified challenges could be grouped into nine themes: ambiguous and inaccurate patient identification, restricted generalisability due to poor geographic transferability of evidence, narrow costing perspective applied, difficulties defining comparators, consequences of applied time horizon, ambiguity in the selection of outcomes, challenged outcome measurement, non-standardised measurement and valuation of costs as well as challenges regarding a reliable preference-based outcome valuation.

**Conclusion:**

Our review offers a comprehensive context-specific overview of methodological considerations for economic evaluations of palliative and end-of-life care. It also identifies the main knowledge gaps to help prioritise future methodological research specifically for this field.

**What is already known about the topic?**

- Palliative and end-of-life care interventions require proof of cost-effectiveness with the help of economic evaluations although some regulatory bodies allow higher cost-effectiveness thresholds.
- There are significant differences between palliative and end-of-life care and other healthcare fields such that some mainstream economic evaluation methods are limited in their suitability.
- There is currently a lack of methodological guidance for conducting economic evaluations in the field of palliative and end-of-life care.

**What this paper adds?**

- This study provides a comprehensive overview of all methodological considerations identified so far in the literature when conducting economic evaluations for adults receiving palliative and/or end-of-life care.
- A 39-point summary has been developed for an easy overview of the main methodological challenges and potential solutions where available.
- This study raises awareness of the necessary considerations for future research design, provides a roadmap for future research and can serve as a basis for developing future methodological guidelines.

**Implications for practice, theory or policy**

- Neglecting context-specific factors during economic evaluations of palliative and end-of-life care can greatly influence the precision of cost-effectiveness results and hinder their comparability.
- The identified themes, namely ambiguous and inaccurate patient identification, restricted generalisability due to poor geographic transferability of evidence, narrow costing perspective applied, difficulties defining comparators, consequences of applied time horizon, ambiguity in the selection of outcomes, challenged outcome measurement, challenges regarding reliable preference-based outcome valuation, non-standardised measurement and valuation of costs, form the starting point for improving the comparability and standardisation of methods applied in future palliative and end-of-life care economic evaluations.
- Challenges relating to patient identification and outcome measurement in economic evaluations of palliative and end-of-life care are intricately connected to and cannot easily be separated from clinical issues.

## Introduction

Over the past century, there has been a significant decline in sudden deaths, resulting in a shift towards a growing population that is living longer and experiencing advanced stages of incurable chronic conditions as they approach the end of life.^1^ Ensuring a high quality of life and dignified death for these individuals poses new challenges for healthcare systems worldwide.^2,3^ In the light of new (expensive) medications and interventions, effective and efficient resource allocation is of great importance in the field of palliative and end-of-life care.^4^ Palliative care, which enhances the quality of life for patients and their families grappling with the challenges of life-threatening illnesses, encompasses end-of-life care as a crucial facet, particularly when patients are nearing the end of life.^5,6^ The costs of care in the last year of life have been estimated to make up 25%– 30% of all medical expenditure during a lifetime^7,8^ and are expected to rise even higher.^9^ Therefore, research is required providing evidence for decisions on clinical guidelines and services.^10^

Economic evaluations, which analyse the costs and consequences of different courses of action,^11^ are widely used to assess the cost-effectiveness of healthcare interventions and to support decision making.^12^ There are two main approaches to conducting economic evaluations: modelling studies and economic evaluations alongside clinical trials. Modelling studies are rare in the palliative and end-of-life care field because of limited data availability, short follow-up times and the need to make numerous assumptions due to the individual nature of patients’ disease courses and experiences.^13,14^ Conducting economic evaluations alongside clinical trials, specifically randomised controlled trials, often presents methodological challenges, including the issue of missing data, particularly within the context of palliative and end-of-life care.^15^ Consequently, relatively few economic evaluations have been conducted in the palliative and end-of-life care field^16,17^ and among those that have been performed, only a small proportion are full cost-effectiveness studies.^18,19^ When conducted, such studies often involve high costs and limited generalisability and do not generate the necessary evidence to inform decision making effectively.^20^ Ongoing discussions are revolving around the potential influence of setting-specific methodological aspects for economic evaluations of palliative and end-of-life care. These settings differ from other healthcare domains and pose unique challenges to mainstream economic evaluation methods. For example, palliative and end-of-life care interventions focus on the quality of dying and broader well-being, rather than extending life, which is not captured by the generic quality-of-life measures that are usually applied.^21,22^ Further, the role of relatives, which is particularly significant in this field,^23,24^ is often overlooked in economic evaluations.^25,26^ It is crucial to consider these context-specific conditions when designing economic evaluations in the palliative and end-of-life care field to ensure that valuable und useful evidence is generated for decision making. Therefore, in the EU project ‘iLIVE – Live well, die well’, in which two palliative and end-of-life care economic evaluations will be executed, we aim to incorporate context-specific recommendations in our development of a methodological framework^27^ as such guidance is otherwise scarce. The evidence-based guidance on the best methods for the design and execution of evaluative end-of-life care research provided by the authors of The Methods Of Researching End of Life Care (MORECare) statement, for example, only includes a few aspects regarding cost-effectiveness. These are ‘integration into preliminary evaluations and testing feasibility of methods’, ‘taking a societal approach when assessing care costs’ and ‘justification of appropriate outcome measures to generate cost-effectiveness’.^20^ Therefore, the primary objective of this study was to conduct a systematic and comprehensive analysis of the literature to identify common methodological aspects and challenges encountered in economic evaluations of palliative and end-of-life care. Another aim was to consolidate and integrate existing recommendations and solutions addressing the challenges identified in order to enhance the methodological framework for future economic evaluations in this field.

## Methods

The study was a protocol-based systematic review (PROSPERO CRD42020148160) drafted in accordance with the reporting guidance provided by the Preferred Reporting Items for Systematic Reviews and Meta-Analyses (PRISMA) 2020 guidelines (Supplemental Tables 1 and 2 in the Appendix).^28^ Additionally, we have published a protocol paper that provides detailed information on our methods.^29^

### Search strategy

We collaborated with an information specialist to review and develop tailored search strings for methodological aspects of palliative care health economics. These strings combined MeSH terms and free text words and were adapted for various databases (Supplemental Table 3 in the Appendix). Our search was limited to English, German, Dutch, French or Spanish articles. We conducted searches in MEDLINE, EMBASE, Cochrane Library, NHS EE and screened relevant websites, including health economic associations and HTA bodies, for grey literature such as guidelines and reports. The search covered articles published from 1 January 1999 to 31 December 2019, with an update on 5 June 2023. Additionally, we retrieved articles from reference lists and recommendations from the iLIVE consortium.

### Screening and eligibility

Publications were imported to EndNote X8, where duplicates were removed. Two screening rounds were performed (title/abstract then full article) using the inclusion criteria outlined in Table 1. Any disagreements were resolved within the research team. To assess screening reliability, the kappa statistic value was calculated. The review included multiple study designs and various quality appraisal tools were applied accordingly, such as the JBI critical appraisal checklist for case reports, text and opinion papers.^30^

**Table 1.** Eligibility criteria.

Population	Adults, 18 years and older with - palliative care (‘. . .*an approach that improves the quality of life of patients and their families facing the problems associated with life-threatening illness, through the prevention and relief of suffering by means of early identification and impeccable assessment and treatment of pain and other problems, physical, psychosocial, and spiritual’*^5^) or - end-of-life care (as a form of palliative care, when the patient is close to the end of life^6^) needs in any care setting (e.g. hospitals, nursing homes, hospices or patients’ own homes). In cases where studies did not define the terms ‘palliative care’ or ‘end-of-life care’ in the publication, the decision to include them was determined based on the described treatment goal for the patient population. Studies were included if it was evident from their description that they focused on a patient population where the main aim was symptom reduction. When the primary aim had a curative intent, the study was excluded. No restrictions were applied based on underlying diseases or patient characteristics.
Study design	A range of study types were considered focusing on the methodology of economic evaluations in the field of palliative and end-of-life care. These included systematic reviews (including meta-analyses), narrative reviews, observational or interventional studies, discussions and commentaries (including editorials), economic guidelines and checklists as well as qualitative studies.
Outcome	Methodological aspects and/or recommendations for conducting economic evaluations in palliative and end-of-life care described in the literature.

### Data extraction, assessment and analysis

We used standardised data extraction forms to gather information from the publications we included, like bibliographical information and study design details. Narrative synthesis, a method used in qualitative research, was used to summarise the information in the articles.^31^ We reviewed the studies and systematically collected information regarding the type of me-thodological aspects described. Our focus was on understanding the specific methodological challenges, the potential impact of these challenges on the evaluation results and any recommended solutions. The relevant information was discussed by the authors, coded and then categorised into overarching descriptive themes. We investigated similarities and differences between the findings by theme and examined the effect of possible determinants, such as study type or investigated patient population, on the reported information. The identified themes were assigned to the reporting items specified in the Consolidated Health Economic Evaluation Reporting Standards (CHEERS) statement, a standard guide for reporting economic evaluations and ensuring their identification, interpretability and usefulness.^32^ This process was guided by the recommendations provided by the Cochrane Consumers and Communication Review Group.^33^

## Results

### Search results

**Figure 1.**
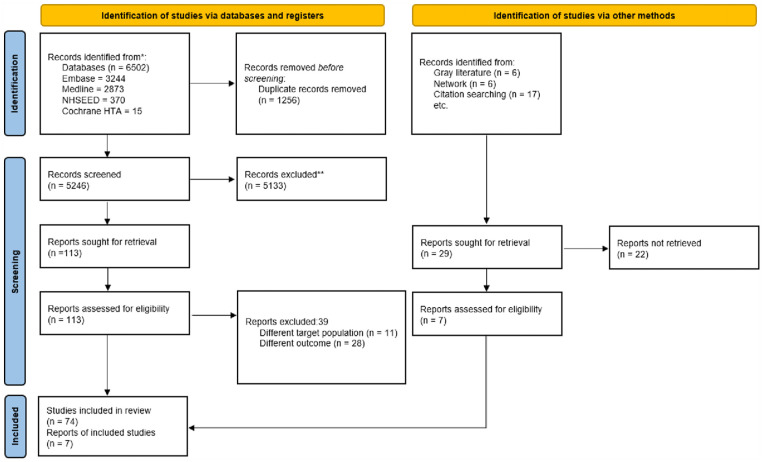
PRISMA 2020 flow diagram.

Figure 1, the PRISMA flowchart, summarises the search process. Out of the 6531 initial references, 81 papers met our inclusion criteria with 98% inter-rater agreement. The papers included reported on both palliative care and end-of-life care, unless explicitly stated otherwise.

### Study characteristics

Supplemental Table 4 (Appendix) summarises the main characteristics of the studies included. The majority were published in the past 10 years (*N* = 59 (70%)). The largest group of first authors (41%) was affiliated with institutions in the UK,^15,16,20,22,26,34–61^ followed by the USA (13%).^62–71^ Study types and their focus varied widely, including systematic reviews and meta-analyses,^17,26,41,52–54,72,73^ qualitative studies^35,36,40,44,49,50,51,58,74^ and studies describing, for example, the development and/or concept of an outcome measure, testing its validity. The overall quality of the studies included was high and none was excluded based on low quality appraisal scores.

### Synthesis

In total, we identified challenges that can be grouped into nine themes, with most of the studies relating to three of them: ambiguity in the selection of outcomes,^16,17,20–22,26,34–50,62,63,72,74–88^, non-standardised measurement and valuation of costs,^15,17,26,40–42,47,51–57,61,64,65,73,85,89–93^ and narrow costing perspective app-lied.^17,20,24–26,52,53,62,64,73,76,90,94–96^ Other methodological challenges identified related to ambiguous and inaccurate patient identification,^15,45,67–70,76,85,94,96–99^ restricted generalisability due to poor geographic transferability of evidence,^16,40,42,43,52,53,60,71,85,96,98^ difficulties defining comparators,^15,97^ consequences of applied time horizon,^65^ challenged outcome measurement^35,45,57,89,97,100^ and challenges regarding a reliable preference-based outcome valuation.^37,45,58,59,66,72,84,85,87,101–105^

Table 2 provides an overview of all of the challenges and potential solutions identified in the literature categorised by methodological theme (Themes 1–9). Although our systematic review identified nine groups of challenges, we did not find any information pertaining to the following CHEERS checklist reporting items: discount rate, currency, rational of model, assumptions, characterising heterogeneity, distributional effects and uncertainty or engagement with patients. Detailed discussions of the methodological issues that are of particular importance in economic evaluations are provided in the following subsections (Themes 2, 3, 5, 6, 8 and 9). Three themes, namely challenges related to patient identification (Theme 1), defining comparators (Theme 4) and outcome measurement (Theme 7) in economic evaluations of palliative and end-of-life care are intricately connected to and cannot easily be separated from clinical issues. Given their broad implications beyond economic evaluations, these challenges are included in the overview table (Table 2) for completeness but are not discussed extensively in the results section.

**Table 2.** Overview of methodological challenges and potential solutions identified in the included literature.

Overarching theme	Identified challenges	Identified recommendations/potential solutions
(1) Ambiguous and Inaccurate Patient Identification	Definition Ambiguity & Inaccurate Patient Identification -No standardised way of determining end-of-life onset,^98^ no single source available for accurate prognosis.^98^ -Labelling and communicating terminal status poses challenges, affecting end-of-life trial participation.^15^ -No ICD-10 code for ‘palliative care encounter’.^94^	-Identification via care process, asking patients about their status, refined prognostic criteria models, diagnosis.^45^
		-Professionals’ expectations and prognostic models to refine patient life-expectancy assessment.^98^
	Patient Selection Bias -Concerns about study burden, patient health and perceived intervention benefits may hinder patient recruitment.^45,70,76,85^ -Challenge to reach underrepresented vulnerable populations for research.^70^ -Obstacles in obtaining patients’ informed consent.^15,69^ -Certain therapies have dual palliative and curative purposes^106^ and the timing of initiating specific palliative care services varies widely in the target population.^85^	-Application of population-wide study recruitment using registry data (e.g. the All Patient Revised Diagnosis Related Group (APR-DRG)^18,67,68^ or the AN-SNAP classification system^99^).
		-Before-and-after quasi-experimental study designs may minimise selection bias concerns in concurrent treatment and comparison group studies.^97^
		-Educating staff and patients about the nature of a study, associated time, effort, risks and benefits to alleviate the perceived study burden.^70^
(2) Restricted Generalisability due to Poor Geographic Transferability of Evidence	Restricted Generalisability -due to high care customisation.^16,42,52,71,83,95,105^	-Identification of transferable and locally dependent intervention characteristics.^60^ -Segmentation of heterogeneous palliative care population into more homogeneous groups during study planning.^98^
	Limited Transferability -of results across health care systems (e.g. due to different health care structures).^107^	-Application of comparative data collection tools and adjustment of study design to the given context.^40,53^
(3) Narrow Costing Perspective Applied	Narrow Hospital/Payer/Health Care Perspective -provides biased picture of effects and costs.^47,53,96^	-Adoption of societal perspective.^17,24–26,52,53,76,90,95^
		-Application of two reference cases (health/social care and societal) in cost-effectiveness planes to satisfy diverse information needs.^17,62^
		-Multi-agency cost consequence analyses to assess sector-specific cost components.^53^
(4) Difficulties Defining Comparators	Difficulty in Defining Comparators -Defining comparators in economic evaluations of palliative and end-of-life care is challenging due to: -the holistic and individual nature of the interventions -different treatments under the label of palliative care varying between diagnosis and prognosis.^97^	-Precise documentation is needed of elements of standard care, the intervention tested and the differences between them. -Application of adequate statistical methods (e.g. multivariate models) to deepen understanding of the factors driving the results.^15^
(5) Consequences of Applied Time Horizon	Consequences of Applied Time Horizon -The length of time over which the costs and consequences of the interventions may influence the type of costs considered and the cost saving potential of an intervention.^65^	-The time horizon should align with the intended effects and capture the relevant costs. For example, short-term horizons in ICUs may focus on variable costs like drugs while longer-term horizons require assessing both variable and fixed costs to reflect costs accurately.^65^
(6) Ambiguity in the Selection of Outcomes	It is unclear whether to give Preference to Generic or Context-Specific Outcome Measures.^16,17,45,47,50,62,72,74,76,78,80–82,84,85,87,88^	-Application of context-specific outcome measures.^74,87^
		-Application of outcomes with a narrow focus on the process and aim of the tested intervention.^76^
		-Testing well-established, generic measures along with disease-specific ones.^20,46,48,49,63,74,76,88^
	Neglecting Effects on Relatives although their well-being may be heavily affected by informal care giving.^41,43,82,83^	-Outcome and cost impacts for patients’ relatives must not be overlooked.^44,77,82^ -Assessment needs to be improved^17^ and new assessment tools tested.^86^
7) Challenged Outcome Measurement by Missing Data and no Consensus on Assessment Method	High Attrition Rates and Frequent Unfeasible Direct Assessment -High attrition rates pose a challenge to the measurement of effectiveness.^26^ -Proxy assessment is frequently required due to patients’ frailty and inability to provide data. However, it remains uncertain which source of proxy assessment is the most reliable.^35^	-Rigorous planning and sufficient funding are required.^20,40,41^
		-Design randomised trials adequately powered for economic evaluations.^97^
		-The lack of assessment of self-reported outcomes in standard patient care hinders the ability to link data and conduct broader or secondary analyses.^94^
		-Recent empirical evaluations indicate: -insignificant differences between terminal lung cancer patients’ and nurses’ EQ-5D scores.^89^ -proxy assessment (ICECAP-SCM) seems to be only challenging for respondents with limited . . .knowledge of the patient,^35^ with close friends/family providing the most consistent responses.^100^
		-Further research is needed to assess the feasibility of routine data collection^35^ and to validate the use of proxy assessments in this field.^45^
(8) Challenges Regarding Reliable Preference-Based Outcome Valuation	Values may not Reflect Patient Preferences -Preference-based outcome values are often derived from the general population and not directly from end-of-life patients.^45^	-Values from, for example, care professionals with better insights into the relevance of certain capabilities should be explored.^45^
	Application of Generic Quality-of-Life Instruments -Domains relevant to palliative care may be missed as non-specific quality-of-life measurement tools are commonly used to elicit preferences about dying in palliative care.^84^	-Instruments used to elicit preferences about dying should include essential domains such as ‘preparation for death’ and ‘managing affairs’, as for example demonstrated in the End-of-Life Preferences Interview (ELPI).^105^
	Variation in the valuation of Patient-Reported Outcome Measures (PROMs) -Respondents may assign values to aspects of care that contradict the underlying quality paradigm.^87^ -Patients may choose not to use certain services but put value on their availability.^37,85,87^	-The availability of services alongside actual use of them should be considered in end-of-life care evaluations.^85^ -Evaluating care packages instead of individual components may help detect such phenomena.^87^
(9) Non-Standarised Measurement and Valuation of Costs	Non-Standardised Cost Estimation Methods -due to weak study designs, varying time points of assessment, lack of cross-sectoral data collection.^26,41,42,47,51,53,54,64,85,92,93^	-Alongside a comprehensive reflection on palliative and end-of-life health and social care costs (e.g. for hospice care), the effects on other sectors (so-called ‘spill-over costs’) also need to be considered (e.g. lost productivity).^53,85^
		-Careful consideration of the assessment moment to capture fluctuating costs and longitudinal assessment should start early enough.^16,17,52^
		-Inclusion of affected family network (the three closest individuals at least).^51^
	Cost assessment is challenged by Definition Problems especially regarding informal care(givers), (beginning of) caregiving time and an inability to distinguish costs related to advanced disease and natural deterioration from those related to intervention.^61^	-More reliable longitudinal quantification of informal care-related costs and carer time instead of standard costs using relevant cost-driving factors.^41^
		-Development/application of standardised, unambiguously defined cost items aiming for a comprehensive costing framework covering multiple sectors.^16,42,73^
		-Stronger study designs and novel approaches (e.g. modelling) to explore more accurate cost estimates.^16,53^
		-Local validation of contextually relevant and sensitive data collection tools for reliable data.^41^
	No consensus on Preferred Data Collection Tool for palliative and end-of-life care patient and family costs.^40,41,54^	-Irrespective of the method, consideration of sensitive delivery/participant engagement, matching research questions and engagement building by conducting initial face-to-face interviews.^40^
		-Minimise recall bias by adding specific survey instructions on counting/time frames.^40,56,91^
	Costs Related to Patients’ Absence from Work are insufficiently documented and highly dependent on the valuation method applied.^55,64^	-Early starting, longitudinal cost assessment to capture fluctuating productivity declines.^64^
		-Application of market wages to document net effect of palliative care on work productivity changes.^64^

### Theme 2: Restricted generalisability due to poor geographic transferability of evidence

The generalisability of palliative and end-of-life care economic evaluations may be limited, even with detailed descriptions of the geographical location and the healthcare setting in which the intervention was carried out.^16,71^ Highly customised care delivery, even within disease groups and the influence of local and regional factors (e.g. differences in care teams, religion and cultures)^42,52,83,95,105^ contribute to these limitations. As healthcare system funding varies across countries, the burden on patients and families, as measured by the impact of informal care costs^52^ and healthcare-related out-of-pocket expenses during the last year of life, also varies accordingly. For instance, in Europe, these costs can range from 2% to 25% of median household income.^108^ The transferability of palliative and end-of-life care economic evaluation results to low and middle-income countries is especially limited. Therefore, there is a need for more studies to be conducted directly in these countries^42,52,53^ to assess the applicability of methods.^107^ However, due to poor palliative care development, limited public financing and practical obstacles, such studies are still rare.^85,96^

#### Recommendations

The transferability of results across healthcare systems requires international collaboration,^43,53^ the identification and separation of locally dependent and transferable intervention characteristics^60^ and the stratification of heterogeneous palliative populations into more similar subgroups during study planning.^98^ Standardised data collection methods facilitate international comparative economic data^53^ which would be needed for valid cross-country comparisons of costs and outcomes. These methods should also account for country- or region-specific factors, such as the association between family income and informal caregiving costs.^40^

### Theme 3: Narrow costing perspective applied

Palliative and end-of-life care involves a range of professionals, supplementary services and informal carers.^15,26,47,85^ The costs and effects of such care may not only affect different areas within the healthcare sector but also beyond,^17,53,64,73,90^ encompassing ‘spill-over’ effects^64,94^ such as costs incurred by the employers of palliative care patients.^53,64^ However, a systematic review of palliative and end-of-life care economic evaluations revealed that informal care costs are often neglected. None of the 18 papers included estimated these costs.^109^ This can be attributed to various factors. Many studies tend to focus primarily on costs borne by the provider or the funder.^47,53,96^ This aligns with the perspective recommended in national economic evaluation guidelines that have been slow to incorporate a broader societal perspective (e.g. the UK^110^).

#### Recommendations

To capture impacts across sectors, it is recommended to adopt a societal perspective.^20^ Using this broader costing perspective, which considers costs beyond health and social care sectors, increases understanding of the full cost impact of the intervention, facilitating informed decision making.^17,24–26,52,53,76,90,95^ To accommodate the needs of different groups, it is recommended to employ diverse reference cases in cost-effectiveness planes, using both a health and social care system or payer perspective as well as a broader societal perspective.^17,62^ Furthermore, conducting multi-agency cost consequence analyses enables the assessment and disaggregated presentation of sector-specific cost components.^53^

### Theme 5: Consequences of applied time horizon

The length of time over which the costs and consequences of the interventions are calculated may influence the type of costs considered and the cost saving potential of an intervention^65^

#### Recommendations

The time horizon should align with the intended effects and capture the relevant costs. For example, short-term horizons in ICUs may focus on variable costs like drugs while longer-term horizons require assessing both variable and fixed costs to reflect costs accurately.^65^

### Theme 6 a–b: Ambiguity in the selection of outcomes

The health outcomes measured should reflect patient preferences, meet psychometric criteria^21^ and reflect state-of-health levels.^44,79^ Currently it is unclear which of the following outcomes can fulfil these criteria for palliative and end-of-life care economic evaluations:

- *Generic outcome measures*: Generic quality-of-life measures such as the EQ-5D-5L have broad applicability and comparability across diseases and settings; however, their precision is debatable in certain cases.^35,46^ The treatment focus of palliative and end-of-life care differs from curative care, which raises questions about the validity of using quality-adjusted life years (QALYs), which are based on generic health-related quality-of-life measures, as outcome measures in this field.^16,17,47,62,72,74,76,87,88^ Although these measures may not fully capture the relevant quality dimensions,^84^ they may still produce plausible changes that seem to justify their application, yet still introduce biased results.^85^
- *Context-specific outcome measures*: Several specific patient-reported outcome measures for palliative and end-of-life care have been developed^34,38,39,87^ (e.g. EORTC QLQ-C15-PAL^81^ Palliative Care Outcome SCALE^38,39,87^) but most are unable to generate the preference-based utility weights that are necessary for conducting cost-utility analyses.^39,46,80,96^ Attempts to map sector-specific outcome measures onto established utility measures have been challenging.^39^ While disease-specific outcome measures, such as for cancer, may yield preference-based utility weights, they do not cover the complete spectrum of quality-of-life dimensions pertinent to palliative and end-of-life care.^78,80–82^
*- Other approaches*: Broader capability well-being measures, such as the ICECAP Supportive Care Measure (ICECAP-SCM), provide an alternative approach.^35^ Aligning with the capability approach, these measures concentrate on domains related to achieving a good life and good death for patients and their relatives.^35,45,50^ Although several psychometric validation studies on the ICECAP-SCM have been recently published,^36,48,49,75^ more comparative research is needed in this area.^45,50^ Another measure is the Palliative Care Yardstick, which aims to assign a higher value to end-of-life care^22^ and address dimensions such as caregiver impact^47^ and valuation problems.^37,47,87^ Further development of the measure, including the Valuation Index Palliative, is still pending.^22,42,88^ Even if specific measures capturing all relevant quality metrics are developed, cross-sector comparisons between palliative and curative care remain a challenge.^85,87^

#### Recommendations

Given the difficulty of comparing outcomes between palliative and curative care, the inclusion of context-specific outcome measures is recommended^74,87^ despite the expected heterogeneity. In the absence of an ideal outcome measure specific to palliative and end-of-life care, the application of generic health-related quality-of-life measures is recommended alongside context-specific outcome measures to enable further methodological evaluations.^20,46,48,49,63,74,76,88^ Furthermore, longitudinal assessment of outcome measures with a narrow focus on the process and the specific aim of the intervention, like symptom control, is advised for future studies focusing on palliative and end-of-life care.^76^

### Theme 6 b

Palliative and end-of-life care not only impacts patients and formal caregivers but also their relatives.^43,82^ Only a small percentage of palliative care patients receive hospital inpatient care.^83^ In many developed countries, there is a policy trend towards shifting the provision of palliative and end-of-life care into a community setting.^111,112^ As a result, informal caregivers (i.e. family and friends) take over a considerable share of care and can face significant out-of-pocket expenses. While the burden on informal caregivers is dominant,^56^ they can also experience positive rewarding effects of caregiving, resulting in what is a complex impact overall on their own health and quality of life.^52^ If ignored, these impacts may lead to a biased value assessment of palliative and end-of-life interventions.^41,82^ Although acknowledged, informal caregiver perspectives are currently overlooked in palliative and end-of-life care economic evaluations.^83^

#### Recommendations

There is no consensus on an appropriate methodology for outcome measurement. Outcomes should be measured multi-dimensionally and include the patients’ close relatives. 44, 77, 82 A recent study by Pop et al.^86^ that reviewed instruments used to assess burdens on family caregivers identified the Burden Scale for Family Caregivers as the most useful tool for clinical practice. Applying and comparing different approaches within one study may help to assess their validity and reliability and advance the methodological debate.^17^

### Theme 8 a–b: Challenges regarding reliable preference-based outcome valuation

Current methods to assign a monetary value to preference-based outcome measures (i.e. valuation) by estimating health state ‘utilities’ or willingness to pay, for example, may not fully reflect the perspectives of palliative and end-of-life care patients, consequently leading to a biased interpretation of results. Firstly, preference-based outcome values are not generated from end-of-life patients themselves but from the general population.^45^ Secondly, often quality-of-life measurement tools that are not specific to palliative care are used to elicit preferences about dying.^84^ In fact, a recent review by Quinn et al.^72^ that studied stakeholder preferences for end-of-life care confirmed multiple violations of the underlying assumptions of using QALYs to assess preferences in this context.

#### Recommendations

Although still unexplored, health and care professionals involved in palliative and end-of-life care may have a better understanding of the relevance of certain capabilities of these patients.^45^ It is essential to include the domains ‘preparation for death’ and ‘managing affairs’ in such instruments, as has been done in the end-of-life preferences interview developed by Borreani et al.^105^ Furthermore, considering our limited understanding of patients’ end-of-life preferences, it is imperative for further research to concentrate on formulating an accepted definition of value at the end of life.^72^

### Theme 8 b

Authors’ opinions, study outcomes and national costing guidelines are inconclusive on the appropriateness of assigning higher weight to end-of-life health gains.^63,102,103^ While new approaches have been introduced and more methodological studies conducted,^58,59,66^ overall, empirical support is limited and further research is needed in this field.^101,104^ Furthermore, individuals may value aspects of care in a way that contradicts the underlying quality paradigm. For example, research has shown that the benefit of interventions for people who are in the same circumstances may vary in a way that cannot be explained by their underlying disease or symptoms.^87^ Furthermore, patients may choose not to use certain services although they may place value on their availability.^37,85,87^

#### Recommendations

Normand^85^ suggests considering the availability of services alongside their actual use in end-of-life evaluations. Evaluating packages of care instead of individual components may also help to detect these phenomena more easily.^87^

### Theme 9 a–d: Non-standardised measurement and valuation of resources and costs

Understanding the true costs of palliative and end-of-life care is limited.^53,93^ Mixed results on the costs and cost-effectiveness of palliative care have been published, showing that palliative care can result in lower,^113,114^ equal^115,116^ or higher^117^ costs compared to usual care. Multiple factors may contribute to this, such as non-standardised costs,^42,47^ variation in approaches to estimating the use and costs of resources related to palliative and end-of-life care^47,54^ and the timing of an intervention in a patient’s journey.^26,42,92^ In addition, access to reliable cost information presents a significant challenge^53,93^ as only a few readily available national unit cost data sources exist that provide palliative care cost estimates (e.g. NHS reference costs, UK).^54^ Hospice care costs in particular seem to be widely unavailable and are therefore often disregarded in studies.^53^ The same holds for spill-over costs,^85^ such as the effect on informal care or children’s academic performance.^64,96^ Finally, the real extent of the effect on a patient’s personal network, which consists of eight individuals on average (with three being more closely involved)^51^ is currently not fully represented in economic evaluations.^41^

#### Recommendations

A comprehensive assessment of the health and social care costs of palliative and end-of-life care (e.g. for hospice care) is required. In addition, the effects on other sectors, such as lost productivity and informal care, need to be taken into account. At least three closely involved individuals, which do not need to be close family, should be considered to capture informal care costs.^51^ In general, careful consideration of the assessment moment is necessary. When aiming to assess fluctuating costs, it is recommended to start collecting cost data at an early stage in a disease trajectory and to complement this with a longitudinal assessment over an entire trajectory.^16,17,52^

### Theme 9 b

Various methods exist for assessing resources and costs for palliative and end-of-life care for both patients and their families, such as self-reported data collection tools including interviews, questionnaires or cost diaries. While they enable assessment across different fields, recall bias^54^ and (emotional) burdens^40,89^ are disadvantages described in the literature. In sum, there is currently no consensus on which tool and administration mode are best.^40,41,54^

#### Recommendations

Regardless of the chosen cost data collection method, sensitive delivery and proper participant engagement are advised. Strategies like initial face-to-face interviews incorporating participant preferences for data collection and maintaining ongoing involvement are recommended to enhance data quality.^40^ Furthermore, for retrospective data collection, it is suggested to use a two-week recall period^91^ and to provide survey instructions that emphasise counting tasks only once and considering the stated time frame in activity assessment.^40,56^

### Theme 9 c

Variations in the definitions of cost items can significantly impact analysis outcomes.^92^ Estimating palliative hospital costs becomes challenging when assessing intensive care unit costs, a crucial cost driver. As multiplying the average cost per intensive care unit day by the length of stay may result in an imprecise representation of costs, obtaining data on primary costs per day would provide a more accurate estimation. If unavailable, published estimates of cost variation by day of stay are a recommended alternative.^65^ Nevertheless, distinguishing these costs from other health care costs, such as managing adverse events and providing follow-on treatments, can still be difficult, especially in severe health states characterised by natural deterioration.^61^

#### Recommendations

Valid comparisons across studies, countries and health care systems, require standardised, locally validated and contextually relevant cost items^16,41,73^ and definitions.^42^ Future studies are needed to make progress in validating cost assessment methods.^52^ With stronger study designs and novel methods, such as economic modelling approaches, more accurate cost estimates may be explored.^16,53^ Furthermore, to improve cost assessment, it is vital to increase the reliability when quantifying ‘carer time’, to validate contextually relevant assessment tools and to involve all relevant parties. Gardiner et al.’s^53,55^ framework, which incorporates family costs, is considered the most comprehensive costing framework for palliative care so far but also Urwin et al.^57^ have recently introduced a post-bereavement cost measure of informal end-of-life cancer care, confirming its content validity and feasibility.

### Theme 9 d

The costs of disease-related work absenteeism, essential for understanding productivity fluctuations over time, are insufficiently documented.^64^ Palliative care in an early disease stage may exacerbate declines in productivity due to medical appointments, whereas in later stages, it can help patients to continue doing their job.^64^ Furthermore, attributing a monetary value to work absenteeism is complex as it is highly dependent on the method applied.^55,64^

#### Recommendations

Accurate cost assessment is recommended, valuing productivity changes based on market wages and thoroughly documenting the impact of palliative care on work productivity.^64^

## Discussion

### Summary of main findings

Palliative and end-of-life care is filled with highly personalised experiences and disease progressions; therefore, traditional incremental outcome and cost analyses may have difficulties capturing effects. Our study aimed to systematically and comprehensively identify common methodological challenges of conducting economic evaluations relating to palliative and end-of-life care described in the literature. Further, it aimed to synthesise existing recommendations and potential solutions to overcome the challenges identified. Our systematic review revealed nine themes encompassing the methodological challenges encountered when conducting economic evaluations in the field of palliative and end-of-life care. While certain themes like patient identification and outcome measurement when faced with high dropout rates are commonly acknowledged in evaluative research in this field, the majority of the other themes are unique to economic evaluations in this context. Given our study aim to offer a comprehensive overview of the methodological aspects involved in conducting an economic evaluation and considering that these aspects align with the items outlined in the CHEERS checklist, we decided to include all relevant items in our analysis.

### Implications for research and policy

The strength of the solutions and recommendations identified varied across themes, with some themes having a substantial number of clear recommendations, such as for the ‘narrow costing perspective’ or ‘ambiguity in the selection of outcome measures’; for others, the recommendations were brief and/or provided no concrete solutions. While the discussions regarding the most suitable outcome measurement instrument are ongoing, there has been a noticeable increase in psychometric validation studies focusing on specific tools like the ICECAP-SCM.^48,49,118,119^ This growing body of research provides valuable information about the suitability and validity of such tools. Both in terms of challenges and potential solutions, the list of recommendations is not exhaustive. With the increasing body of methodological research on economic evaluations for palliative and end-of-life care outcomes as well as the growing application of economic evaluations in this context, it is likely that additional recommendations will be added or existing ones will be refined. Findings from other settings should also be considered in palliative and end-of-life care economic evaluations. For instance, the generic self-reported PECUNIA RUM instrument, an internationally standardised, harmonised and validated tool for resource use measurement, can provide a comprehensive picture of resources and costs across various sectors, including health and social care, education, (criminal) justice, productivity losses and informal care from a societal perspective.^120–122^ Furthermore, it is harmonised with other PECUNIA costing tools to achieve cross-country and cross-sectoral comparability in costing methods.^123^ Nevertheless, the challenge of differentiating between the effects of an advanced disease stage and natural disease progression on costs and outcomes alike will remain. These factors may hinder an accurate assessment of the value of palliative and end-of-life care interventions. Fast disease progression in end-of-life patients leads to a natural health status deterioration, complicating the imp-act assessment of an intervention. Additionally, natural health status deterioration in advanced disease stages may result in high resource use and costs unrelated to the intervention.^61^ Our systematic review fills an important gap in the literature by offering an initial overview of methodological considerations in economic evaluations for palliative and end-of-life care. Our findings will inform the economic evaluation of palliative and end-of-life care interventions in the iLIVE project,^27^ guide future evaluations and promote transparency and comparability. In the light of varying methodological approaches to economic evaluations in the palliative and end-of-life care field,^18,124,125^ this list of nine themes is essential as it forms the foundation for prioritising the research agenda and developing comprehensive guidelines for conducting future economic evaluations in the field of palliative and end-of-life care. Further methodological research in this field is imperative. It is essential to channel these efforts towards validating the identified information and refining recommendations, thereby improving the precision and relevance of these guidelines.

### Strengths and limitations

This systematic review has multiple strengths but also limitations. As this systematic review was carried out alongside a large EU project (‘iLIVE – Live well, die well’^27^) in the field of palliative and end-of-life care, we were able to discuss methodological factors with different relevant stakeholders (such as clinicians and researchers) as well as incorporate and test the findings in practice. The broad nature of our research question challenged the development of a targeted search strategy. Therefore, a robust methodology was developed and piloted, including a specific search strategy for multiple electronic databases of peer-reviewed literature and tailored search strings carefully refined for every database developed in cooperation with an information specialist. Further, two individual researchers were involved in the screening and data extraction processes. While the systematic review only covered five languages, this is unlikely to influence the findings since the systematic review had good coverage of countries with a strong track record both in economic evaluations and palliative and end-of-life care. The generalisability of the overall findings, however, should be regarded as limited to high-income countries. For low and middle-income countries, a separate systematic review may be needed. Since authors did not always explicitly define whether they were referring to end-of-life, palliative care or both patient groups and any designations usually lacked clear definitions, a further limitation of our synthesis lies in the restricted possibility of separating aspects relevant only to palliative care, end-of-life care or both. An accurate definition of the patient group addressed has also been identified as one of the methodological recommendations for future palliative and end-of-life care studies.

## Conclusion

Our list of 39 recommendations aims to overcome most of the challenges identified above as well as to improve the comparability and overall transparency and to standardise the methodology and execution of future economic evaluations conducted for palliative and end-of-life care. It also identifies the main knowledge gaps to help prioritise future methodological research specifically for this field. The list has been implemented and is currently being tested within the international iLIVE project but should be generalisable beyond the project.

## Supplemental Material

sj-pdf-1-pmj-10.1177_02692163231214124 – Supplemental material for Methodological challenges and potential solutions for economic evaluations of palliative and end-of-life care: A systematic review Click here for additional data file. Supplemental material, sj-pdf-1-pmj-10.1177_02692163231214124 for Methodological challenges and potential solutions for economic evaluations of palliative and end-of-life care: A systematic review by Claudia Fischer, Damian Bednarz and Judit Simon in Palliative Medicine
