# Supplementary material for: Methodological challenges and potential solutions for economic evaluations of palliative and end-of-life care: A systematic review
Source: Palliat Med. 2023 Dec 23;38(1):85–99. doi: 10.1177/02692163231214124 (PMC10798028; doi:10.1177/02692163231214124)
Supplement: sj-pdf-1-pmj-10.1177_02692163231214124 – Supplemental material for Methodological challenges and potential solutions for economic evaluations of palliative and end-of-life care: A systematic review [file sj-pdf-1-pmj-10.1177_02692163231214124.pdf]

APPENDIX

Table1. PRISMA 2020 checklist

Section and Topic	Item #	Checklist item	Location where item is reported
**TITLE**			
Title	1	Identify the report as a systematic review.	1
**ABSTRACT**			
Abstract	2	See the PRISMA 2020 for Abstracts checklist.	3
**INTRODUCTION**			
Rationale	3	Describe the rationale for the review in the context of existing knowledge.	4, 5
Objectives	4	Provide an explicit statement of the objective(s) or question(s) the review addresses.	5
**METHODS**			
Eligibility criteria	5	Specify the inclusion and exclusion criteria for the review and how studies were grouped for the syntheses.	6
Information sources	6	Specify all databases, registers, websites, organisations, reference lists and other sources searched or consulted to identify studies. Specify the date when each source was last searched or consulted.	6
Search strategy	7	Present the full search strategies for all databases, registers and websites, including any filters and limits used.	6, Appendix
Selection process	8	Specify the methods used to decide whether a study met the inclusion criteria of the review, including how many reviewers screened each record and each report retrieved, whether they worked independently, and if applicable, details of automation tools used in the process.	7
Data collection process	9	Specify the methods used to collect data from reports, including how many reviewers collected data from each report, whether they worked independently, any processes for obtaining or confirming data from study investigators, and if applicable, details of automation tools used in the process.	7
Data items	10a	List and define all outcomes for which data were sought. Specify whether all results that were compatible with each outcome domain in each study were sought (e.g. for all measures, time points, analyses), and if not, the methods used to decide which results to collect.	7

Section and Topic	Item #	Checklist item	Location where item is reported
	10b	List and define all other variables for which data were sought (e.g. participant and intervention characteristics, funding sources). Describe any assumptions made about any missing or unclear information.	7
Study risk of bias assessment	11	Specify the methods used to assess risk of bias in the included studies, including details of the tool(s) used, how many reviewers assessed each study and whether they worked independently, and if applicable, details of automation tools used in the process.	7
Effect measures	12	Specify for each outcome the effect measure(s) (e.g. risk ratio, mean difference) used in the synthesis or presentation of results.	7
Synthesis methods	13a	Describe the processes used to decide which studies were eligible for each synthesis (e.g. tabulating the study intervention characteristics and comparing against the planned groups for each synthesis (item #5)).	7
	13b	Describe any methods required to prepare the data for presentation or synthesis, such as handling of missing summary statistics, or data conversions.	n.a.
	13c	Describe any methods used to tabulate or visually display results of individual studies and syntheses.	7
	13d	Describe any methods used to synthesize results and provide a rationale for the choice(s). If meta-analysis was performed, describe the model(s), method(s) to identify the presence and extent of statistical heterogeneity, and software package(s) used.	7
	13e	Describe any methods used to explore possible causes of heterogeneity among study results (e.g. subgroup analysis, meta-regression).	n.a.
	13f	Describe any sensitivity analyses conducted to assess robustness of the synthesized results.	n.a.
Reporting bias assessment	14	Describe any methods used to assess risk of bias due to missing results in a synthesis (arising from reporting biases).	n.a.
Certainty assessment	15	Describe any methods used to assess certainty (or confidence) in the body of evidence for an outcome.	n.a.
**RESULTS**			
Study selection	16a	Describe the results of the search and selection process, from the number of records identified in the search to the number of studies included in the review, ideally using a flow diagram.	8
	16b	Cite studies that might appear to meet the inclusion criteria, but which were excluded, and explain why they were	Figure 1

Section and Topic	Item #	Checklist item	Location where item is reported
		excluded.	
Study characteristics	17	Cite each included study and present its characteristics.	Appendix, Table 4
Risk of bias in studies	18	Present assessments of risk of bias for each included study.	n.a.
Results of individual studies	19	For all outcomes, present, for each study: (a) summary statistics for each group (where appropriate) and (b) an effect estimate and its precision (e.g. confidence/credible interval), ideally using structured tables or plots.	n.a.
Results of syntheses	20a	For each synthesis, briefly summarise the characteristics and risk of bias among contributing studies.	n.a.
	20b	Present results of all statistical syntheses conducted. If meta-analysis was done, present for each the summary estimate and its precision (e.g. confidence/credible interval) and measures of statistical heterogeneity. If comparing groups, describe the direction of the effect.	n.a.
	20c	Present results of all investigations of possible causes of heterogeneity among study results.	n.a.
	20d	Present results of all sensitivity analyses conducted to assess the robustness of the synthesized results.	n.a.
Reporting biases	21	Present assessments of risk of bias due to missing results (arising from reporting biases) for each synthesis assessed.	n.a.
Certainty of evidence	22	Present assessments of certainty (or confidence) in the body of evidence for each outcome assessed.	n.a.
**DISCUSSION**			
Discussion	23a	Provide a general interpretation of the results in the context of other evidence.	20
	23b	Discuss any limitations of the evidence included in the review.	21
	23c	Discuss any limitations of the review processes used.	21
	23d	Discuss implications of the results for practice, policy, and future research.	20, 21
**OTHER INFORMATION**			
Registration and protocol	24a	Provide registration information for the review, including register name and registration number, or state that the review was not registered.	6

Section and Topic	Item #	Checklist item	Location where item is reported
	24b	Indicate where the review protocol can be accessed, or state that a protocol was not prepared.	6
	24c	Describe and explain any amendments to information provided at registration or in the protocol.	6
Support	25	Describe sources of financial or non-financial support for the review, and the role of the funders or sponsors in the review.	23
Competing interests	26	Declare any competing interests of review authors.	23
Availability of data, code and other materials	27	Report which of the following are publicly available and where they can be found: template data collection forms; data extracted from included studies; data used for all analyses; analytic code; any other materials used in the review.	n.a.

Table 2. The PRISMA for Abstracts Checklist

Section and Topic	Item #	Checklist item	Reported (Yes/No)
**TITLE**			
Title	1	Identify the report as a systematic review.	2
**BACKGROUND**			
Objectives	2	Provide an explicit statement of the main objective(s) or question(s) the review addresses.	2
**METHODS**			
Eligibility criteria	3	Specify the inclusion and exclusion criteria for the review.	2
Information sources	4	Specify the information sources (e.g. databases, registers) used to identify studies and the date when each was last searched.	2
Risk of bias	5	Specify the methods used to assess risk of bias in the included studies.	2
Synthesis of results	6	Specify the methods used to present and synthesise results.	2
**RESULTS**			
Included studies	7	Give the total number of included studies and participants and summarise relevant characteristics of studies.	2
Synthesis of results	8	Present results for main outcomes, preferably indicating the number of included studies and participants for each. If meta-analysis was done, report the summary estimate and confidence/credible interval. If comparing groups, indicate the direction of the effect (i.e. which group is favoured).	2
**DISCUSSION**			
Limitations of evidence	9	Provide a brief summary of the limitations of the evidence included in the review (e.g. study risk of bias, inconsistency and imprecision).	n.a.
Interpretation	10	Provide a general interpretation of the results and important implications.	2
**OTHER**			
Funding	11	Specify the primary source of funding for the review.	Listed in referred protocol and main text
Registration	12	Provide the register name and registration number.	2

Table 3. Search syntax for Medline (OVID)

1. Palliative Care/
2. exp Terminal Care/
3. Terminally Ill/
4. palliat*.mp.
5. (terminal* adj6 (care or caring or ill or illness*)). ti, ab, ot, kf.
6. (end of life or last year of life or lyol or life’s end). ti, ab, ot, kf.
7. advanced cancer. ti, ab, ot, kf.
8. Hospices/
9. hospice*.ti,ab,ot,kf.
10. bereave*.ti,ab,ot,kf,hw.
11. 1 or 2 or 3 or 4 or 5 or 6 or 7 or 8 or 9 or 10
12. exp *Health Care Costs/
13. ((health care or healthcare) adj3 cost*).ti,ot,kf,kw.
14. *‘Costs and Cost Analysis’/
15. Cost-Benefit Analysis/mt [Methods]
16. exp models, economic/
17. (economic* adj3 (evaluat* or aspect* or health or analy* or model* or framework* or frame work* or method*)). ti, ab, ot, kf, hw.
18. economics. ti, ot, kf.
19. Palliative Care/ec
20. exp Terminal Care/ec
21. Hospices/ec
22. 12 or 13 or 14 or 15 or 16 or 17 or 18 or 19 or 20 or 21
23. 11 and 22
24. recycl*.ti,hw,kf,jw.
25. (waste or life cycle assessment). jw.
26. 24 or 25
27. 23 not 26
28. limit 27 to (dutch or english or german or french or spanish)
29. limit 28 to ‘all child (0 to 18 years)’
30. limit 29 to ‘all adult (19 plus years)’
31. 29 not 30
32. 28 not 31
33. limit 32 to yr=‘1999–2023’

EMBASE.com
#33 #28 NOT #31 AND [1-1-1999]/sd NOT [07-06-2023]/sd
#32 #28 NOT #31
#31 #29 NOT #30
#30 #29 AND ([young adult]/lim OR [adult]/lim OR [middle aged]/lim OR [aged]/lim OR [very elderly]/lim)
#29 #28 AND ([adolescent]/lim OR [child]/lim OR [fetus]/lim OR [infant]/lim OR [newborn]/lim OR [preschool]/lim OR [school]/lim)
#28
#23 NOT #26 AND ([dutch]/lim OR [english]/lim OR [french]/lim OR [german]/lim OR [spanish]/lim)
#27 #23 NOT #26
#26 #24 OR #25
#25 waste:jt OR 'life cycle assessment':jt
#24 recycl*:ti,de,kw,jt
#23 #13 AND #22

#22 #14 OR #15 OR #16 OR #17 OR #18 OR #19 OR #20 OR #21
#21 (('health care' OR healthcare) NEAR/3 cost*):ti,kw
#20 economics:ti,kw
#19 (economic* NEAR/3 (evaluat* OR aspect* OR health OR analy* OR model* OR framework* OR 'frame work*' OR method*)):ti,ab,kw
#18 'health care cost'/exp/mj
#17 'economics'/mj
#16 'economic model'/de
#15 'health economics'/de
#14 'economic evaluation'/de
#13 #1 OR #2 OR #3 OR #4 OR #5 OR #6 OR #7 OR #8 OR #9 OR #10 OR #11 OR #12
#12 bereave*:de,ti,ab,kw
#11 hospice*:de,ti,ab,kw
#10 'advanced cancer':ti,ab,kw
#9 'end of life':de,ti,ab,kw OR 'last year of life':de,ti,ab,kw OR 'lyol':de,ti,ab,kw OR 'life s end':de,ti,ab,kw
#8 (terminal* NEAR/6 (care* OR caring OR ill OR illness* OR patient*)):ti,ab,kw
#7 palliat*:ti,ab,kw
#6 'hospice'/de
#5 'terminal disease'/de
#4 'palliative therapy'/exp
#3 'terminal care'/exp
#2 'terminally ill patient'/exp
#1 'palliative nursing'/de

EBM Reviews - Health Technology Assessment Database (OVID) (discontinued at the end of 2016)
1 Palliative Care/
2 exp Terminal Care/
3 terminally ill/
4 palliat*.mp.
5 (terminal* adj6 (care or caring or ill or illness*)).mp.
6 (end of life or last year of life or lyol or life* end).mp.
7 advanced cancer.mp.
8 hospices/
9 hospice*.mp.
10 bereave*.mp.
11 1 or 2 or 3 or 4 or 5 or 6 or 7 or 8 or 9 or 10
12 exp Health Care Costs/
13 ((health care or healthcare) adj3 cost*).mp.
14 "Costs and Cost Analysis"/
15 Cost-Benefit Analysis/mt [Methods]
16 exp models, economic/
17 (economic* adj3 (evaluat* or aspect* or health or analy* or model* or framework* or framework* or method*)).mp.
18 economics.mp.
19 12 or 13 or 14 or 15 or 16 or 17 or 18
20 11 and 19
21 limit 20 to "all child (0 to 18 years)"
22 limit 20 to (dutch or english or german or spanish or french)

EBM Reviews - NHS Economic Evaluation Database (OVID) (discontinued on 31 March 2015)
1 Palliative Care/
2 exp Terminal Care/
3 terminally ill/

4 palliat*.mp. [mp=title, text, subject heading word]
5 (terminal* adj6 (care or caring or ill or illness*)).mp. [mp=title, text, subject heading word]
6 (end of life or last year of life or lyol or life* end).mp. [mp=title, text, subject heading word]
7 advanced cancer.mp. [mp=title, text, subject heading word]
8 hospices/
9 hospice*.mp. [mp=title, text, subject heading word]
10 bereave*.mp. [mp=title, text, subject heading word]
11 1 or 2 or 3 or 4 or 5 or 6 or 7 or 8 or 9 or 10
12 limit 11 to "all child (0 to 18 years)"
13 limit 12 to "all adult (19 plus years)"
14 12 not 13
15 11 not 14
16 limit 15 to yr="1999 - 2015"

Table 4. Characteristics of included studies

Author	Year	Title	Journal	Country (1st author)	Study type	Main focus								
						Inaccurate patient identification	Restricted generalisability due to poor geographic evidence transferability	Narrow costing perspective applied	Difficulties defining comparators	Consequences of applied time horizon	Ambiguity in the selection of outcomes	Challenged outcome measurement	Challenges regarding a reliable preference-based outcome valuation	Non-standardised measurements and valuation of costs
**Al-Janabi et al.** [34]	2008	What do people value when they provide unpaid care for an older person? A meta-ethnography with interview follow-up.	Social science & medicine	UK	Measure development/ Qualitative study						x			
**Andersson et al.** [90]	2002	The economic burden of informal care	International Journal of Technology Assessment in Health Care	Sweden	Case study			x						x
**Antunes et al.** [73]	2018	Outcome measurement-a scoping review of the literature and future developments in palliative care clinical practice	Annals of Palliative Medicine	Portugal	Scoping review			x						x

**Bailey et al.** [35]	2016	The ICECAP-SCM tells you more about what I'm going through: A think-aloud study measuring quality of life among patients receiving supportive and palliative care.	Palliative Medicine	UK	Qualitative study						x	x		
**Bailey et al.** [36]	2018	Hospice patients' participation in choice experiments to value supportive care outcomes	BMJ Supportive & Palliative Care	UK	Qualitative study						x			
**Bhattarai et al.** [59]	2019	The value of dementia care towards the end of life – A contingent valuation study	International Journal of Geriatric Psychiatry	UK	Contingent valuation study								x	
**Bickel et al.** [62]	2017	Importance of costs and cost effectiveness of palliative care	Journal of Oncology Practice	USA	Editorial, review			x			x			
**Boni-Saenz et al.** [64]	2005	The price of palliative care: toward a complete accounting of costs and benefits	Clinics in Geriatric Medicine	USA	Methodological paper			x						x
**Borreani et al.** [105]	2008	Eliciting individual preferences about death: development of the End-of-Life Preferences Interview	J Pain Symptom Manage	Italy	Development of measure								x	
**Brouwer et al.** [77]	2006	The CarerQol instrument: a new instrument to measure care-related quality of life of informal caregivers for use in economic evaluations	Quality of Life Research	The Netherlands	Concept paper of measure /case study						x			
**Canaway et al.** [44]	2017	Development of a measure (ICECAP-Close Person Measure) through qualitative methods to capture the benefits of end-of-life care to those close to the dying for use in economic evaluation	Palliative Medicine	UK	Qualitative study						x			
**Canaway et al.** [51]	2019	Close-Person Spill-Overs in End-of-Life Care: Using Hierarchical Mapping to Identify Whose Outcomes to Include in Economic Evaluations	Pharmacoeconomics	UK	Qualitative study									x

**Chai et al.** [24]	2014	The magnitude, share and determinants of unpaid care costs for home-based palliative care service provision in Toronto, Canada	Health and social care in the community	China/ Canada	Prospective cohort study			x						
**Coast J.** [45]	2014	Strategies for the economic evaluation of end-of-life care: making a case for the capability approach	Expert Review of Pharmacoeconomics & Outcomes Research	UK	Expert review	x					x	x	x	
**Coast et al.** [58]	2020	"It is not a scientific number it is just a feeling": Populating a multi-dimensional end-of-life decision framework using deliberative methods	Health Economics	UK	Qualitative study / Focus groups								x	
**Coast et al.** [46]	2018	Patient centered outcome measurement in health economics: beyond EQ-5D and the Quality-Adjusted Life-Year-where are we now?	Annals of Palliative Medicine	UK	Opinion paper						x			
**Costa et al.** [78]	2014	The development of cancer-specific multi-attribute utility instruments from the EORTC QLQ-C30 and FACT-G	Asia-Pacific Journal of Clinical Oncology	Australia	Development of measure						x			
**Davis et al.** [79]	2017	Quality of life in palliative care	Expert review of quality of life in cancer care	USA	Narrative review						x			
**Davis et al.** [67]	2002	The business of palliative medicine--Part 2: The economics of acute inpatient palliative medicine	American Journal of Hospice & Palliative Medicine	USA	Letter	x								
**Davis et al.** [68]	2004	End-of-life care costs. Journal of Palliative Medicine	Journal of Palliative Medicine	USA	Review/Discussion paper/perspective	x								
**Davison et al.** [95]	2008	Methodological considerations for end-of-life research in patients with chronic kidney disease	Methods and science in nephrology	Canada	Review/Discussion paper/perspective			x						
**Douglas et al.** [37]	2005	A new approach to eliciting patients' preferences for palliative day care: the choice experiment method	Journal of Pain & Symptom Management	UK	Choice experiment						x		x	

**Dumont et al.** [91]	2010	Measurement challenges of informal caregiving: A novel measurement method applied to a cohort of palliative care patients	Social Science & Medicine	Canada	Methodological paper									x
**Dzingina et al.** [39]	2017	Does the EQ-5D capture the concerns measured by the Palliative care Outcome Scale? Mapping the Palliative care Outcome Scale onto the EQ-5D using statistical methods	Palliative Medicine	UK	Cross-sectional study						x			
**Dzingina et al.** [38]	2017	Development of a Patient-Reported Palliative Care-Specific Health Classification System: The POS-E	The Patient: Patient-Centered Outcomes Research	UK	Development of measure						x			
**Eagar et al.** [99]	2004	An Australian casemix classification for palliative care: technical development and results	Palliative Medicine	Australia	Development of case-mix classification	x								
**Eckermann S.** [80]	2017	Health economics from theory to practice	Book	Australia							x			
**Engelberg et al.** [69]	2006	Measuring the quality of dying and death: methodological considerations and recent findings	Current Opinion in Critical Care	USA	Review/Discussion paper/perspective	x								
**Evans et al.** [26]	2013	“Best practice” in developing and evaluating palliative and end-of-life care services: A meta-synthesis of research methods for the MORECare project	Palliative Medicine	UK	Systematic review			x			x			x
**Gardiner et al.** [52]	2014	Exploring the financial impact of caring for family members receiving palliative and end-of-life care: a systematic review of the literature	Palliative Medicine	UK	Systematic review		x	x						x
**Gardiner et al.** [40]	2016	Methodological considerations for researching the financial costs of family caregiving within a palliative care context	BMJ Supportive &B Palliative Care	UK	Qualitative study (semi-structured interviews)		x				x			x

**Gardiner et al.** [41]	2016	Approaches to capturing the financial cost of family care-giving within a palliative care context: a systematic review	Health & Social Care in the Community	UK	Systematic review						x			x
**Gardiner et al.** [53]	2017	What cost components are relevant for economic evaluations of palliative care, and what approaches are used to measure these costs? A systematic review	Palliative Medicine	UK	Systematic review		x	x						x
**Gardiner et al.** [54]	2018	What is the cost of palliative care in the UK? A systematic review	BMJ supportive & palliative care	UK	Systematic review									x
**Gardiner et al.** [55]	2019	Costs of Family Caregiving in Palliative Care (COFAC) questionnaire: development and piloting of a new survey tool	BMJ supportive & palliative care	UK	Development of measure									x
**Gomes et al.** [16]	2009	Optimal approaches to the health economics of palliative care: report of an international think tank	Journal of pain and symptom management	UK	Review/Discussion paper/perspective		x				x			x
**Grande et al.** [15]	2000	Why are trials in palliative care so difficult?	Palliative Medicine	UK	Review/Discussion paper/perspective	x			x					
**Groenvold et al.** [81]	2006	The development of the EORTC QLQ-C15-PAL: a shortened questionnaire for cancer patients in palliative care	European Journal of Cancer	Denmark	Development of measure						x			
**Gühne et al.** [75]	2021	Valuing end-of-life care: translation and content validation of the ICECAP-SCM measure	BMC Palliative Care	Germany	Content validation study						x			
**Guerriere et al.** [25]	2011	The ambulatory and home care record: a methodological framework for economic analyses in end-of-life care	Journal of Aging Research	Canada	Review/Discussion paper/perspective			x						
**Hansen et al.** [104]	2019	Disentangling public preferences for health gains at end-of-life: Further evidence of no support of an end-of-life premium	Social Science & Medicine	Denmark	Web-based survey / stated preferences study								x	

**Harding et al.** [42]	2009	Research priorities in health economics and funding for palliative care: views of an international think tank	Journal of Pain & Symptom Management	UK	Review/Discussion paper/perspective		x				x			x
**Haycox A.** [60]	2009	Optimizing decision making and resource allocation in palliative care	J Pain Symptom Management	UK	Review/Discussion paper/perspective		x							
**Higginson et al.** [43]	1999	Services, costs and appropriate outcomes in end of life care	Annals of Oncology	UK	Editorial		x				x			
**Higginson et al.** [26]	2013	Evaluating complex interventions in End of Life Care: the MORECare Statement on good practice generated by a synthesis of transparent expert consultations and systematic reviews	BMC Medicine	UK	Multi-method approach			x			x			
**Hoefman et al.** [82]	2015	Measuring caregiver outcomes in palliative care: a construct validation study of two instruments for use in economic evaluations	Quality of Life Research	The Netherlands	Construct validation study						x			
**Johnston et al.** [76]	2017	Economics of palliative care: measuring the full value of an intervention	Journal of palliative medicine	Ireland	Editorial	x		x			x			
**Khandelwal et al.** [65]	2017	Evaluating the Economic Impact of Palliative and End-of-Life Care Interventions on Intensive Care Unit Utilization and Costs from the Hospital and Healthcare System Perspective	Journal of palliative medicine	USA	Special report					x				x
**Lakdawalla et al.** [66]	2021	Health technology assessment with diminishing returns to health: Generalized risk-adjusted cost-effectiveness (GRACE) approach	Value in Health	USA	Methodological paper								x	
**Larsson et al.** [100]	2004	Advanced home care: patients' opinions on quality compared with those of family members	Journal of Clinical Nursing	Sweden	Prospective observational study							x		
**Lavergne et al.** [98]	2011	Exploring generalizability in a study of costs for community-based palliative care	Journal of Pain and Symptom Management	Canada	Case study (Secondary database analysis)	x	x							

**May et al.** [17]	2014	Economic impact of hospital inpatient palliative care consultation: Review of current evidence and directions for future research	Palliative Care Review	Ireland	Meta-review			x			x			x
**May et al.** [92]	2016	Analyzing the Impact of Palliative Care Interventions on Cost of Hospitalization: Practical Guidance for Choice of Dependent Variable	Journal of Pain & Symptom Management	Ireland	Simulation study									x
**May et al.** [83]	2017	Current state of the economics of palliative and end-of-life care: A clinical view	Palliative Medicine	Ireland	Review/Discussion paper/perspective						x			
**May et al.** [94]	2018	Economic outcomes in palliative and end-of-life care: current state of affairs	Annals of Palliative Medicine	Ireland	Review/Discussion paper/perspective	x		x				x		
**May et al.** [97]	2020	Economics of palliative care for cancer: interpreting current evidence, mapping future priorities for research	Journal of Clinical Oncology	Ireland	Review	x			x			x		
**McCaffrey et al.** [84]	2009	Measuring impacts of value to patients is crucial when evaluating palliative care	Journal of Pain & Symptom Management	Australia	Letter						x		x	
**McCaffrey et al.** [96]	2015	Bringing the economic cost of informal caregiving into focus	Palliative Medicine	Australia	Editorial	x	x	x						
**McCaffrey & Eckermann** [21]	2018	Raise the Bar, Not the Threshold Value: Meeting Patient Preferences for Palliative and End-of-Life Care	Pharmacoecon Open	Australia	Editorial						x			
**Mosoiu et al.** [93]	2014	Developing a costing framework for palliative care services	Journal of pain and symptom management	Romania	Methodological paper									x
**Murtagh et al.** [47]	2013	Capturing activity, costs, and outcomes: The challenges to be overcome for successful economic evaluation in palliative care	Progress in Palliative Care	UK	Methodological paper						x			x
**Myring et al.** [48]	2022	An analysis of the construct validity and responsiveness of the ICECAP-SCM capability	BMC Palliative Care	UK	Construct validity study						x			

		wellbeing measure in a palliative hospice setting												
**Normand C.** [87]	2009	Measuring outcomes in palliative care: limitations of QALYs and the road to PalYs	Journal of pain and symptom management	Ireland	Methodological paper						x		x	
**Normand C.** [85]	2012	Setting priorities in and for end-of-life care: Challenges in the application of economic evaluation	Health Economics	Ireland	Review/Discussion paper/perspective	x	x				x		x	x
**Nwankwo et al.** [49]	2022	A think-aloud study of the feasibility of patients with end-stage organ failure completing the ICECAP-SCM	Palliative Medicine	UK	Qualitative study						x			
**O'Mara** [70]	2009	Challenges to and lessons learned from conducting palliative care research	Journal of pain and symptom management	USA	Survey	x								
**Pop et al.** [86]	2022	Instruments to assess the burden of care for family caregivers of adult palliative care patients	International Journal of Palliative Nursing	Romania	Narrative review						x			
**Quinn et al.** [72]	2022	No time to waste: An appraisal of value at the end of life	Value in Health	Canada	Systematic review						x		x	
**Reckers-Droog et al.** [101]	2021	Willingness to pay for health-related quality of life gains in relation to disease severity and the age of patients	Value in Health	The Netherlands	Contingent-valuation study								x	
**Reckers-Droog et al.** [102]	2021	Willingness to pay for quality and length of life gains in end of life patients of different ages	Social Science & Medicine	The Netherlands	Contingent-valuation study								x	
**Retzler et al.** [61]	2019	The impact of increased post-progression survival on the cost-effectiveness of interventions in oncology	ClinicoEconomics and Outcomes Research	UK	Case study									x
**Round J.** [22]	2012	Is a QALY still a QALY at the end of life?	Journal of Health Economics	UK	Review/Discussion paper/perspective						x			
**Rowland et al.** [56]	2017	The contributions of family care-givers at end of life: A national post-bereavement census survey of cancer carers' hours of care and expenditures	Palliative Medicine	UK	Survey									x

**Santos et al.** [63]	2022	Guidelines for utility measurement for economic analysis: The Brazilian policy	Value in Health Reg Issues	USA	Methodological study						x			
**Sutton et al.** [50]	2014	Development of a supportive care measure for economic evaluation of end-of-life care using qualitative methods	Palliative Medicine	UK	Qualitative study						x			
**Takura et al.** [89]	2022	Proxy responses regarding quality of life of patients with terminal lung cancer: preliminary results from a prospective observational study	BMJ Open	Japan	Prospective observational study							x		x
**Urwin et al.** [57]	2021	The monetary valuation of informal care to cancer decedents at end-of-life: Evidence from a national census survey	Palliative Medicine	UK	Cross-sectional survey									x
**Weiss et al.** [103]	2020	Therapy preferences in melanoma treatment-Willingness to pay and preference of quality versus length of life of patients, physicians, healthy individuals and physicians with oncological disease	Cancer Medicine	Germany	Survey								x	
**Wichmann et al.** [88]	2017	The use of Quality-Adjusted Life Years in cost-effectiveness analyses in palliative care: Mapping the debate through an integrative review	Palliative Medicine	NL	Integrative review						x			
**Wichmann et al.** [74]	2020	QALY-time: experts' view on the use of the quality-adjusted life year in cost-effectiveness analysis in palliative care	BMC Health Services Research	NL	Qualitative study						x			
**Yang et al.** [71]	2012	Palliative care for the terminally ill in America: the consideration of QALYs, costs, and ethical issues	Medicine, Health Care & Philosophy	USA	Discussion		x							
